# Micro-PEMS Based on OBD and MOX Sensors

**DOI:** 10.3390/s26144333

**Published:** 2026-07-08

**Authors:** Jordy Alexander Hernández, José Ignacio Huertas

**Affiliations:** Sustainable Energy Research Group, School of Engineering and Sciences, Tecnológico de Monterrey, Monterrey 64849, Mexico

**Keywords:** real driving conditions, tailpipe emissions, near-continuous monitoring

## Abstract

**Highlights:**

**What are the main findings?**

- This work reports the development of a proof-of-concept µPEMS that continuously reports in real time the mass emissions of gas phase pollutants (CO, NO_x_) and greenhouse gases (CO_2_), highly correlated with the ones obtained with a 1065-compliant PEMS, when monitoring road vehicles working under normal conditions of use.
- Metal oxide (MOX) sensors can be used for the direct measurement (without gas pre-treatment) of tailpipe air pollutants. Their cross-sensitivity problems can be resolved using a multilinear correlation with the factors influencing them. The signal drift in pollutant concentration can be mitigated by reporting cumulative mass emissions rather than emission rates.

**What are the implications of the main findings?**

- The µPEMS can provide valuable data on the real vehicular emissions that could be used for improving vehicle technology and national emission inventories. It could also enable new alternatives to regulate vehicular emissions. However, additional work is required to explore the possibility of using other sensors and to evaluate the performance of the µPEMS after long hours of service. Low-cost, data drift, and cross-sensitivity are the main issues to be resolved.

**Abstract:**

In response to the EURO 7 regulation, which mandates near-continuous monitoring of pollutant gas emissions from every vehicle during real driving conditions, this research reports the development of a micro portable emissions monitoring system (µPEMS) for monitoring tailpipe mass emissions of NO_x_, CO, and CO_2_. It consists of low-cost MOX sensors installed in the exhaust pipe to detect pollutant concentrations, complemented with engine operation data from the vehicle’s On-Board Diagnostics (OBD) system. Issues of sensor drift, cross-sensitivity, and varying sampling frequency were addressed. Readings from this µPEMS prototype exhibited high correlation (*R*^2^ > 0.87) with experimental data obtained under real driving conditions using a well-accepted PEMS for the cases of three vehicles (gasoline, diesel, and hybrid). This innovation enables new alternatives to regulate vehicular emissions. It also provides valuable real-time data for improving ecodriving, vehicle technology, and national emission inventories.

## 1. Introduction

Currently, there is an urgent need for an instrumental system (µPEMS), permanently embedded in each vehicle, that continuously measures tailpipe mass emissions and periodically uploads the data to the cloud. The primary pollutants of interest are carbon monoxide (0–5%), nitrogen oxides [NO (0–5000) ppm and NO_2_ (0–2500) ppm], and particulate matter (PM_1_, PM_2.5_), which must be measured across wide concentration ranges under varying temperature and humidity conditions.

This system will enable drivers, fleet managers, and governmental authorities to monitor and report vehicular emissions in near-real time with high accuracy. Considering that CO_2_, CH_4_, and N_2_O are greenhouse gases (GHGs) present in tailpipe emissions, the µPEMS could also support emission inventories and track progress towards NDC (Nationally Determined Contribution) commitments of GHG reductions [1]. Furthermore, the µPEMS could become an easy and low-cost alternative for regulating vehicular emissions, especially in heavy-duty vehicles, which remain under-regulated in most countries. The automotive industry needs this kind of system to demonstrate the improved emission conformity demanded by regulations, such as EU VII [2]. This system would also enable the handling of emission zones [3,4]. Finally, this system can detect the effects of simultaneous faults in the catalytic converter, lambda probe, or injector, which can lead to excessive emissions [5,6].

Despite the widespread adoption of electric vehicles in urban centers, several applications, such as long-distance transportation and off-road operations, will continue to rely on fuel-powered vehicles [7]. To date, vehicle emissions remain the primary source of air pollutants in large urban centers [8]. In an effort to reduce air pollution in these areas, environmental authorities have established regulations targeting brand-new vehicles that mandate CVS (constant volume sampler) laboratory tests, in which vehicles should comply with threshold-limiting values for mass tailpipe emissions [9]. Over time, these regulations have become progressively more restrictive, resulting in significantly cleaner vehicles compared to previous years [10]. The process of fulfilling these regulations involves costly equipment and high operating costs [11]. As a result, few countries have the facilities to enforce those types of regulations under their local conditions, and therefore, most countries rely on manufacturer tests [12].

Several studies have indicated that vehicles emit significantly more pollutants under real driving conditions than reported by manufacturers, primarily due to differences between real-world and laboratory test conditions [13]. To address these discrepancies, researchers developed portable emission monitoring systems (PEMSs) to measure vehicle emissions by on-road tests [14]. The US 40 CFR 1065 regulates measurement techniques, methods, and verification processes that PEMSs can use [15]. PEMSs were also incorporated into regulations in Europe for testing real driving emissions [16]. AVL, Sensors, Inc., and Horiba are among the most popular trademarks for 1065-compliant PEMSs. All PEMSs use NDIR sensors for the determination of HC, CO, and CO_2_ concentration, as well as the chemiluminescence (CLD) or non-dispersive ultraviolet (NDUV) methods for the determination of NO and NO_2_. Thus, sensors that measure pollutant concentration do not exhibit cross-sensitivity, but they do exhibit signal drift, which limits testing time to ~1 h before recalibration. PEMSs also include an exhaust flow meter (EFM) to measure gas mass flow using Pitot or Venturi tubes. PEMSs have minor data synchronization issues because the two devices (sensors and EFMs) are in the same position.

The investment and operational costs of PEMSs are also high. Their size and weight are still significant. They are delicate and require specialized personnel for their operation. Those circumstances make them invasive and unsuitable for continuous operation over extended periods. Consequently, results from on-road tests using PEMSs are obtained under controlled conditions during short-term tests, failing to capture emissions as the vehicle operates under everyday normal conditions. Ideally, each vehicle should be equipped with a PEMS that continuously monitors its emissions. Additionally, it should feature an information technology system that periodically reports cumulative real driving emissions to relevant stakeholders. PEMSs should therefore be significantly smaller, cheaper, and more user-friendly than current PEMSs [4,17].

The idea of a small-sized PEMS (mini-PEMS) that could be used for non-regulatory purposes evolved more than 10 years ago. Maha developed a mini-PEMS that measures NO_x_, CO_2_, and PM. The company 3DATX developed their parSYNC mini-PEMS that includes measurement of NO_x_, CO_2_, and PM mass [18]. NGK Spark Plug developed their NTK Compact Emissions Meter (NCEM) to measure PM, particle number (PN), NO_x_, O_2_, and air/fuel ratio [19]. These mini-PEMSs use different commercially available sensor technologies [20]. Some of them use electrochemical cells or the NO_x_ dissociation technique to measure NO and NO_2_. These mini-PEMSs also rely on the EFM used by PEMSs to measure mass gas flow, making them still too costly and heavy to be used permanently in each vehicle. They also require frequent recalibration to counteract signal drift. Ref. [21] compared a mini-PEMS with a 1065-compliant PEMS under road conditions and found high correlation in their results. They suggested that the mini-PEMS could be used as a screening tool to measure a large number of vehicles operating under a wide range of conditions.

Some authors have suggested the use of instant fuel consumption and air–fuel ratio to determine mass gas flow at the exhaust in place of the EFM [21,22,23,24]. This alternative significantly reduces the cost and volume of mini-PEMSs because these two pieces of information are already available from the engine control unit (ECU) and can be retrieved via the OBD port. We will refer to them as a micro-PEMS (µPEMS). Ref. [22] suggested a self-contained unit temporarily installed at the tailpipe, powered by an independent energy source and capable of data acquisition/transmission independent of the vehicle’s OBD, that could be used for temporary applications, e.g., for estimating the exhaust gas quality in the control procedure of the Periodical Inspection in Germany (TÜV), MoT in England, and Inspection and Maintenance I/M in the USA. However, they did not demonstrate the device’s operation. Ref. [23] presented an exploratory study carried out with a µPEMS of reduced size (45 × 30 × 20 cm) and weight (~15 kg) for applications on two-wheeler vehicles. They measured the exhaust gas concentrations of HC, CO, and CO_2_ with the NDIR method, and NO_2_ and O_2_ using electrochemical cells. Electrochemical cells require gas pretreatment (drying and particle filtering) for their proper operation. They obtained high correlations between the results obtained with their µPEMS and the one obtained with a PEMS under laboratory conditions.

In this work, we propose to advance previous works and take advantage of the OBD system and advances in sensor technology to address the need for low-cost µPEMS. The objective of the present work is to demonstrate the feasibility of µPEMS based on MOX sensors and OBD data for the continuous, real-time measurement of tailpipe emissions from road vehicles. It is out of the scope of the present work to measure particulate matter, CH_4_, or N_2_O.

In the process of achieving this objective, we reached the following contributions to new knowledge: (i.) the development of a proof-of-concept µPEMS that continuously reports in real time the mass emissions of gas phase pollutants (CO, NO_x_, CO_2_), highly correlated with the ones obtained with a 1065-compliant PEMS, when monitoring road vehicles working under normal conditions of operation; (ii.) additionally, the use of metal oxide (MOX) sensors for the direct measurement (without gas pretreatment) of tailpipe concentration and the solution of their cross-sensitivity problems by the use of a multilinear correlation with their influencing factors; and (iii.) finally, an alternative to mitigate signal drift in pollutant concentration by reporting cumulative mass emissions rather than emission rates.

## 2. Materials and Methods

**Figure 1:**
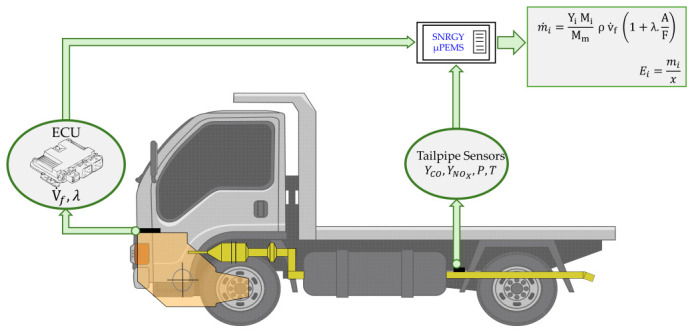
Illustration of the working principle of the proposed µPEMS based on OBD data and MOX sensors.

The working principle of the μPEMS consists of determining exhaust mass flow through the readings of fuel consumption ($\dot{vf}$) and air–flow ratio (AF) from the OBD system, and then multiplying it by the mass fraction ($X_{i}$) of the pollutant of interest, using independent sensors installed directly at the exit of the tailpipe without requiring any gas pretreatment. The proposal is summarized in Figure 1.

This section describes the operation of the µPEMS and their calibration process. First, the selection and setup of a multi-gas sensor to measure pollutant concentration at tailpipe conditions are explained. Subsequently, the process of gathering data from the OBD system is described. Then, the method to calculate pollutant mass emissions is presented. Finally, the work conducted to calibrate the µPEMS is shown.

### 2.1. Sensors to Measure CO and NO_x_ Concentrations at Tailpipe Conditions

The mass concentration of each pollutant *i* at the tailpipe conditions is determined through Equation (1), where *Y_i_* is the volumetric concentration of pollutant *i*, and *M* and *M_i_* are the molecular weights of the combustion products and pollutant *i*, respectively. *Y_i_* is the only variable in Equation (1).

(1) $$X_{i}=Y_{i}\frac{M_{i}}{M}$$

Thus, the µPEMS requires sensors to measure the instant CO and NO_x_ volumetric concentration ($Y_{i}$) at the exit of the vehicle tailpipe in the ranges of 0–5.0% and 0–0.5%, respectively. CO_2_ can be determined directly from fuel consumption. Particulate matter sensors are of great importance for the µPEMS, but they are excluded from the scope of the present work due to the additional challenges they pose. Pollutant concentration must be measured at high temperatures (200–400 °C), in the presence of other pollutants, and under high humidity (15–100%) in a pulsating flow at near-atmospheric pressure [25]. Low energy consumption, low cost, and portability are essential characteristics these sensors must meet to be incorporated into the µPEMS.

Table 1 lists sensors currently available that could be used to measure tailpipe pollutant concentrations. Among them, metal oxide (MOX) semiconductors exhibit the most promising performance for the current application. They are the least expensive detectors available commercially.

#### 2.1.1. MOX Sensors

The surface of the MOX sensor layer consists of small-grained ceramics (e.g., metal-doped SnO) as the gas-sensitive material. Detailed composition and grain diameter yield different gas sensitivities that depend on the manufacturer. When exposed to tailpipe gases, molecules of the gas stream are adsorbed at the ceramic surface as they interact with the ceramic’s oxygen atoms, thereby varying the material’s electrical resistivity. In the case of the 3A4P-UST Triplesensor, the temperature can be controlled with a platinum heater to improve the absorption process, thereby increasing its gas sensitivity [33,34]. Exposure of the MOX material to H_2_O, CO, CH, and NH_3_ decreases its electrical resistance, while exposure to NO and NO_2_ will increase its resistance. Exposure to CO_2_ and N_2_O does not influence the resistance of the material due to their non-reactive behavior. The electrical resistance of the MOX sensor should be divided by the value reported by the same sensor when the pollutant concentration is zero. The logarithm of this fraction is proportional to the pollutant concentration. MOX sensors present two issues: cross-sensitivity and drift.

- Cross-sensitivity refers to the fact that the sensor is sensitive to the presence of multiple gases (e.g., CO, H_2_O, several CH, NH_3_, and NO_x_) [33,34]. Various studies reported that the application of MOX sensors in automotive gas mixtures exhibits cross-sensitivity, particularly influenced by humidity and temperature, leading to depletion problems, [35] and resulting in inaccurate readings [36,37]. In MOX technology, sensitivity can be improved by operating with thermal modulation and employing multiple selective layers [38,39]. Additionally, sensor arrays combined with multivariable analysis can mitigate cross-sensitivity, enabling the detection of the target gas within a mixture of gases [40,41,42].
- Drift is defined as the gradual, time-dependent variation in the sensor’s bulk conductivity due to prolonged use and exposure to corrosive gases [43]. Long-term evaluations of calibrated sensor arrays have demonstrated a substantial reduction in gas-recognition performance, declining from 98% to 20% over three years [44]. It has been found that the rate of change in this conductivity, when driven by a pulsed input, is more stable and reproducible [45]. Thus, to avoid sensor drift over time, periodic recalibration, or the application of temperature control to the imaginary part of the sensing layer impedance, has been proposed [39,46,47]. Additionally, it can be solved by adjusting the measurements, using the CO_2_ concentration measurements obtained by other sensors or methods as a reference.

**Figure 2:**
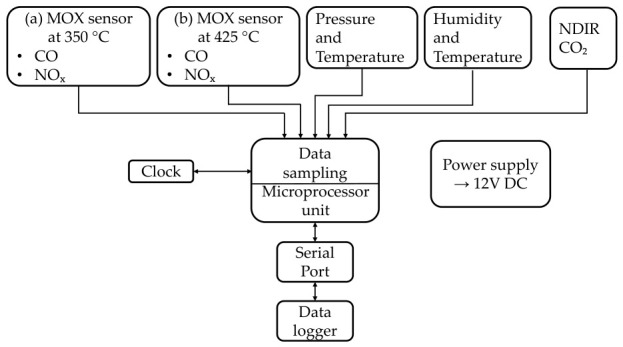
Block diagram of the embedded system for the measurement of CO, NO*_x_*, and CO*_2_* concentrations at tailpipe conditions based on MOX sensors.

Umweltsensortechnik (UST) [48] provides sensors with three layers of MOX materials for measuring CO, NO_x_, and HC in each layer. Deckma Hamburg [49], a partner company, integrated two MOX sensors operating at different temperatures (350 and 425 °C), along with CO_2_, humidity, temperature, and pressure sensors in a homemade multi-gas-sensor module. It includes (Figure 2) a battery-assisted temperature-corrected clock and a 12 V DC power supply. Data are transmitted via RS-232 serial link at 115 kbaud. It was built on a PCB (printed circuit board) with dimensions of 6 × 10 cm^2^, and a measuring area with a diameter of 2.5 cm, which is exposed to exhaust gas via a copper connection pipe. Supplementary details of this configuration can be found in [50,51]. Additional work was undertaken to adapt the output of this device to a portable datalogger, enabling automatic data collection and recording during on-road tests. We named this version of the µPEMS as the MOX-µPEMS.

#### 2.1.2. Zirconia-Based Electrochemical Sensors

Zirconia-based electrochemical sensors have been used for oxygen monitoring and combustion control. These solid-state devices are highly robust and are currently implemented in nearly all internal combustion engines where they supply feedback for air–fuel ratio control. Through modifications in sensor architecture and operating principles, this technology has been extended to enable the detection of NO_x_ at ppm concentrations, supplying critical input for the control and optimization of exhaust post-treatment systems [52]. In this work, we also used commercially available NO_x_ sensors manufactured by Bosch. A data acquisition system was developed to enable online readings from this sensor. We named this version of the µPEMS as NO_x_-µPEMS.

### 2.2. Measurement of Exhaust Mass Emission Rate

Usually, existing PEMS determine the mass exhaust rate ($\dot{m}$) by multiplying the volumetric flow of combustion products in the exhaust pipe ($\dot{\;v_{p\;}}$) with their density (${\;\rho }_{p}$). These instruments use either Pitot or Venturi tubes to measure the volumetric flow of combustion products, and pressure and temperature sensors to measure density.

Alternatively, we propose obtaining the mass exhaust rate ($\dot{m}$) through Equation (2), where $\rho_{f}$ is the fuel density, $\dot{\;v_{f\;}}$ is the volumetric fuel consumption rate, (1 − λ) is the excess air, and *AF* is the air–fuel stoichiometric ratio. All these variables are constant except for $\dot{v_{f\;}}$ and λ, which need to be continuously measured. We propose to read these variables directly from the Engine Computer Unit (ECU) by using an LM327 OBD-II adapter connected to the vehicle’s OBD port. The adapter sends data to the cloud via the internet or to a computer via Bluetooth at a frequency of 1 Hz.

(2) $$\dot{m}=\rho_{f}\dot{\;v_{f\;}}\left(1+\lambda \;AF\right)$$

### 2.3. Determination of the CO and NO_x_ Mass Emissions Rates

Finally, the mass emission rate of pollutant *i* is determined by multiplying the mass fraction of pollutant *i* by the exhaust mass emissions rate ($\dot{m}$) as per Equation (3), which results in Equation (4).

(3) $$\dot{m_{i}}=X_{i}\dot{m}$$

(4) $$\dot{m_{i}}=Y_{i}\frac{M_{i}}{M}\rho_{f}\dot{\;v_{f\;}}\left(1+\lambda \;AF\right)$$

However, implementing Equations (3) or (4) has three complications: sensors have different response times, data are gathered at different sampling frequencies, and data are unsynchronized.

#### 2.3.1. Differences in Sampling Frequency

A recurrent problem that arises when measuring the tailpipe mass emissions is the variation in sensors’ time response and sampling frequencies of the signals involved. These issues are resolved by computing the average value of each variable over the same window time. Although we targeted average mass emissions at 1 Hz, we found that the best results are obtained at lower frequencies. These analyses will be presented in Section 3.

#### 2.3.2. The Time-Alignment Problem

Synchronization problems arise from the fact that some variables (e.g., fuel consumption) are measured at the engine, while others (e.g., pollutant concentration) are measured several meters downstream at the tailpipe exit. Even then, although measurements are taken simultaneously, data are asynchronized. The delay time mostly corresponds to the time it takes for the combustion byproducts to travel from the engine to the tailpipe. This time depends on the exhaust mass flow. Furthermore, differences in the diffusivity of different pollutants cause the delay time to differ between pollutants. We adopted the dynamic data synchronization proposed by [53] to resolve this issue. It consists of shifting one signal forward or backward with respect to the signal that should be synchronized (e.g., fuel consumption and CO_2_ concentration). The delay time ($\Delta t_{\left(t\right)}$) follows Equation (5), where $t_{o}$ and β are constant.

(5) $$\Delta t_{\left(t\right)}=t_{o}+\beta \frac{1}{\dot{v_{f\;}}}$$

A correlation analysis between the signals involved (CO_2_ concentration and fuel consumption, as in our previous example) will be used to determine the level of synchronization. Thus, the constants in Equation (5) are the ones that maximize the coefficient of determination (*R*^2^). This proposal was verified with a large number of tests (>240) carried out with different PEMSs and several diesel- and gasoline-powered vehicles (>70) [53].

### 2.4. Calibration by On-Road Tests

As mentioned previously, the focus of this work is the development of a “proof-of-concept” µPEMS by using existing sensors rather than developing new sensors. Therefore, the demonstration of its functionality must be based on on-road tests comparing its results with those obtained with 1065-compliant PEMSs. Thus, the two versions of the µPEMS were mounted on diesel, gasoline, and hybrid gasoline–electric (HEV) vehicles. Table 2 describes the technical characteristics of the vehicles used in the tests. The test vehicles were pick-ups and SUVs from recent model years.

**Figure 3:**
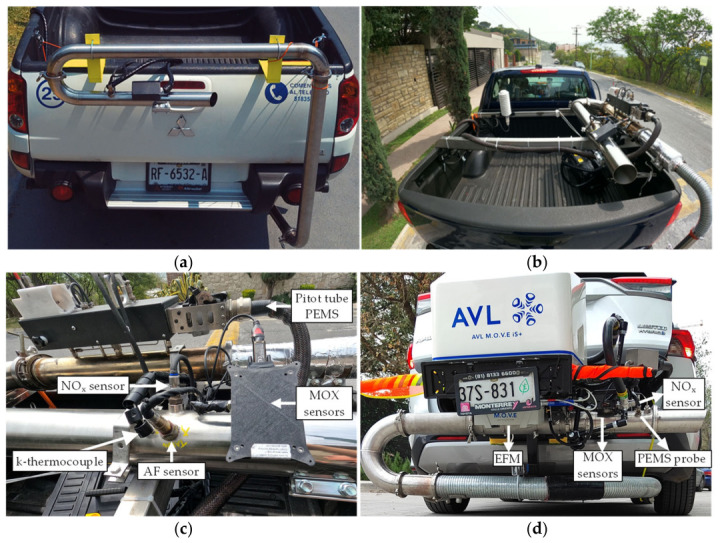
Installation of measurement equipment in the test vehicles. (**a**) General view of the measurement system in a diesel vehicle; (**b**) general view of the measurement system in a gasoline vehicle; (**c**) detailed view of each sensor; (**d**) detailed view of the measurement system in a HEV.

Assembling the µPEMS onto the vehicle required developing an exhaust gas cooling system to ensure temperatures remained below 200 °C upon contact with the µPEMS. To accomplish this objective, the exhaust pipe was extended 2 m with a stainless-steel pipe. Sensors were assembled to avoid exposure to condensed water from combustion products. Figure 3c shows the location of these sensors in the exhaust pipe extension.

Calibration: Measurements from the MOX-µPEMS and NO_x_-µPEMS were intercompared with measurements from a 1065-compliant PEMS. Table 3 presents the technical specifications of the PEMS used for intercomparison. It was the AVL MOVE iS+ PEMS. It was mounted in the vehicles in accordance with the manufacturer’s instructions. Traceable NIST calibration gases were used prior to and after each test, following the operational recommendations provided by the PEMS manufacturer.

**Figure 4:**
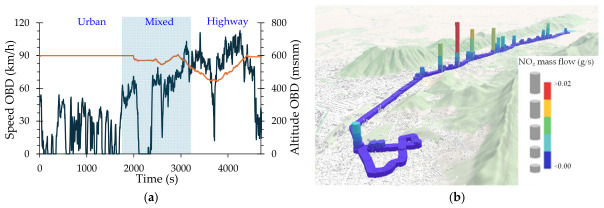

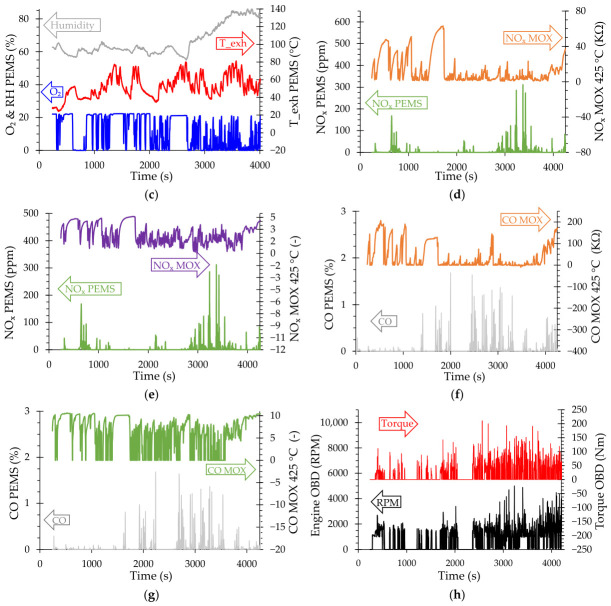
Measured variables during the on-road tests in a HEV. (**a**) OBD measurements for speed (dark green line) and altitude (orange line). (**b**) Location (latitude, longitude), including NO_x_ emissions measured by the PEMS, plotted on the city map where the RDE test was conducted. (**c**) Oxygen (bright blue line), relative humidity (gray line), and exhaust gas temperature (red line) were measured by the PEMS. (**d**) NO_x_ concentrations obtained with the PEMS (light green line) and with the MOX sensor (light orange line). (**e**) NOx concentrations obtained with the PEMS (light green line) and with the MOX sensor after applying a natural logarithm to the data (purple line). (**f**) CO concentrations measured by the PEMS (gray line) and by the MOX sensor (orange line). (**g**) CO concentrations measured by the PEMS (gray line) and by the MOX sensor after applying a natural logarithm to the data (green line). (**h**) Engine RPM (black line) and torque (red line).

The tests were conducted by driving the vehicles along the main roads of the city of Monterrey, Mexico, following existing traffic, while all the variables described above were simultaneously recorded at one-second intervals. Figure 4a shows the profiles of speed and altitude. The tests lasted ~90 min and covered ~70 km.

## 3. Results

Figure 4 shows the results obtained during one of the road tests. It shows the results obtained with the hybrid vehicle.

### 3.1. Data Synchronization and Averaging Time Window

Given the disparity in sampling frequencies across the measurement systems installed on the vehicle (PEMS, NO_x_ sensor, MOX sensor, and OBD system data), it was necessary to unify these to a common frequency prior to further analysis. This down-sampling (with appropriate windowing and filtering) mitigates biases from uneven temporal sampling and differences in sensor response times, as well as reducing noise.

**Figure 5:**
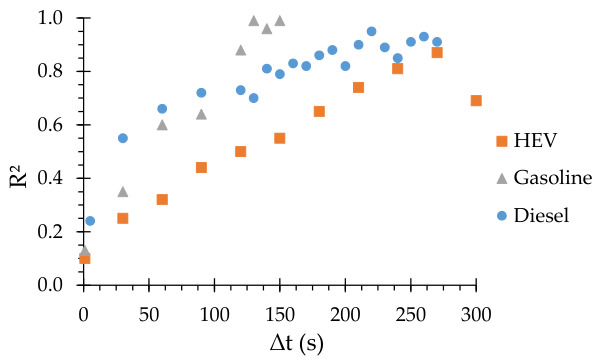
Determination of average time window and data synchronization between fuel consumption, NO_x_, and CO emissions for gasoline, diesel, and HEVs.

Simultaneously, data synchronization was performed at each time averaging frequency. Figure 5 presents, for illustrative purposes, the variation in correlation coefficients (*R*^2^) between the fuel consumption rates and CO_2_ mass emission rates as a function of the time averaging frequency. The best results were observed when the sampling rate averaged every 140 s for gasoline vehicles, 220 s for diesel vehicles, and 270 s for HEVs.

### 3.2. Performance of the MOX Sensor

**Figure 6:**
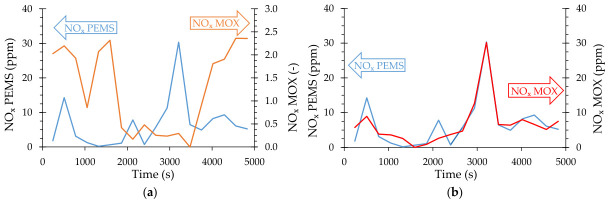
HEV test. The time window *t* (s) < 5000 was considered for the analysis. (**a**) Concentration profiles of the NO_x_ PEMS as the reference instrument (blue line) and the natural logarithm of the NO_x_-sensitive layer (orange line). (**b**) NO_x_ concentration profiles measured by the PEMS reference instrument (blue line) and by the MOX sensor after applying the linear combination model (red line).

As mentioned above, the logarithm of the output of the NO_x_-sensitive layer of the MOX sensor, heated to 425 °C, should be proportional to the NO_x_ concentration. In this case, we used the NO_x_ concentration measured by the PEMS as the reference. Figure 6a shows the evolution of both signals, illustrating that they are highly uncorrelated (*R*^2^ ~ 0.054) in the HEV case. Similar results were observed in the other vehicles.

As described in Section 2.1, the NO_x_ concentration from the MOX sensor can be obtained as a linear combination of the multiple variables that influence its response. Thus, we proposed a multiple linear combination of the variables influencing the MOX sensor response (Equation (6)). That is, the *C_i_* coefficients of Equation (6) need to be determined to predict the NO_x_ concentration. In this equation, rH represents the relative humidity measured by the humidity sensor; T is the temperature measured by the humidity sensor; $P_{t}$ denotes the temperature measured by the pressure sensor; P corresponds to the absolute pressure measured by the pressure sensor; $ln\left(CH/CH_{ref}\right)$ expresses the natural logarithm transformed resistance of the MOX $CH_{s}^{\prime }$ sensing layer; $ln\left(CO/CO_{ref}\right)$ refers to the natural logarithm transformed resistance of the MOX CO sensing layer; and $ln\left(NO/NO_{ref}\right)$ captures the natural-log-transformed resistance of the MOX NO sensing layer.

(6) $$Y_{NOx}=C_{1}+C_{2}\mathrm{rH}+C_{3}T+C_{4}\mathrm{Pt}+C_{5}P+C_{6}ln\left(\frac{CH}{{CH}_{ref}}\right)+C_{7}\;ln\left(\frac{CO}{CO_{ref}}\right)+C_{8}\;ln\left(\frac{NO}{NO_{ref}}\right)$$

Since, in practice, only the signals reported by the multisensory device will be available, the regression was limited to the variables it can read. In this case, we used one MOX sensor heated to 350 °C and another to 425 °C. Table 4 presents the results of the correlation analysis conducted for one of the time windows considered. In this case, a high adjusted coefficient of determination (*R*^2^ > 0.87) was observed in the three vehicles (Figure 6b). The *C_i_* coefficients obtained for Equation (6) are also shown in Table 4. Finally, this table shows the *p*-values for each coefficient. *p-*Values > 0.05 indicate variables that are not relevant in the correlation and can therefore be excluded from Equation (6). Readings of NO_x_ from the second MOX sensor showed a *p-*value < 0.05 and therefore could be excluded from Table 4. The fact that the second reading from MOX showed a *p-*value < 0.05 indicates that these readings are correlated with the readings from the first MOX sensor.

Previous results indicate that the MOX sensor measures NO_x_ concentrations comparable to those obtained with the PEMS and the zirconia-based electrochemical NO_x_ sensor. Similar results were obtained in tests with gasoline-powered vehicles and diesel-powered vehicles.

### 3.3. Performance of the Zirconia-Based Electrochemical NO_x_ Sensor

**Figure 7:**
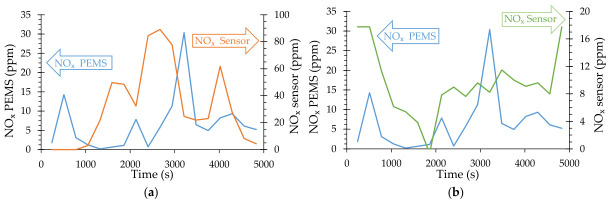
HEV test. Performance of the zirconia-based electrochemical NO_x_ sensor. (**a**) Concentration profiles of the NO_x_ PEMS as the reference instrument (blue line) and the zirconia-based electrochemical NO_x_ sensor (orange line); (**b**) concentration profiles of the NO_x_ PEMS as the reference instrument (blue line) and the zirconia-based electrochemical NO_x_ sensor after applying the linear combination model (green line).

The zirconia-based electrochemical sensor used to measure NO_x_ concentration in the tailpipe was included in the experimental setup used during the tests conducted with the HEV, with the aim of determining its performance relative to the PEMS. Figure 7a shows that the amperometric sensor tends to overestimate NO_x_ concentration and exhibits response delay issues. Therefore, both signals exhibit a poor coefficient of determination (*R*^2^ of 0.008).

However, when the sensor was subjected to the calibration procedures described in Section 2—including corrections for the time-alignment problem and differences in sampling frequency—an improvement in the readings was observed, increasing the *R*^2^ to 0.54; these results are illustrated in (Figure 7b). Although the resulting *R*^2^ remains lower than expected, this outcome is understandable considering that such sensors are primarily designed to evaluate NO_x_ in heavy-duty vehicle technologies, where emissions are significantly higher. Consequently, when applied to technologies such as HEVs, which are characterized by low NO_x_ emissions, their performance is limited.

### 3.4. Determination of Mass Emissions

Measurements taken during the on-road test were processed using the methodologies described above to obtain the mass emission rates of NO_x_ and CO. Next, we will concentrate on the tests conducted with the gasoline hybrid vehicle.

**Figure 8:**
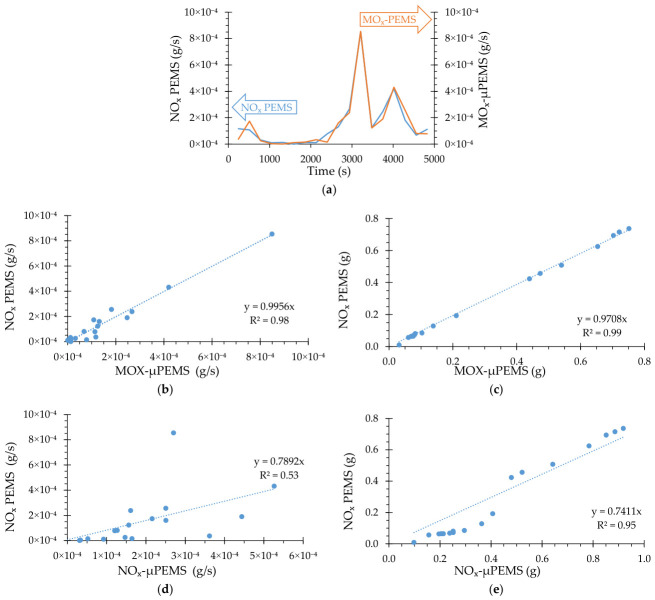
On-road NO_x_ mass emissions of HEVs. (**a**) NO_x_ mass flow rate monitored by the PEMS (blue line) and the MOX-µPEMS (orange line). (**b**) Correlation of NO_x_ mass flow rate: PEMS vs. MOX-µPEMS. (**c**) Correlation of accumulated NO_x_ mass: PEMS vs. MOX-µPEMS. (**d**) Correlation of NO_x_ mass flow rate (MOX-µPEMS vs. PEMS). (**e**) Correlation of accumulated NO_x_ mass, with NO_x_-µPEMS on the *x*-axis and PEMS on the *y*-axis.

*Results in terms of mass flow rate of pollutants*: Figure 8a illustrates the evolution of the NO_x_ mass flow rate, obtained with averaging time windows of Δ*t* = 270 s, during an arbitrary segment of the on-road test. It shows that the MOX-µPEMS produce results that follow the PEMS measurements of NO_x_ mass flow rate. Figure 8b confirms this qualitative result. It shows that the MOX-µPEMS produced highly correlated results (*R*^2^ = 0.98) of NO_x_ mass flow rate compared with the reference PEMS. The slope close to one is a result of the calibration process. Similar results were obtained for the case of NO_x_-µPEMS (Figure 8d).

*Results in terms of cumulative mass emission of pollutants:* To determine the monitored cumulative mass emission, previous mass emission rate results were integrated over time. Again, results were compared with those obtained with the reference PEMS. Figure 8c shows that they are even more correlated (*R*^2^ = 0.99) than in the previous case. This observation was expected, as integrating the variables over time damps errors that frequently occur when sampling time-dependent physical processes. Similar results were obtained for the case of NO_x_-µPEMS (Figure 8e).

*Results in terms of emission index:* Emission indices were obtained by integrating the mass emission rate (mg/s) over time and by normalizing the distance traveled (mg/km) after frequency harmonization and data synchronization. Table 5 shows the average emission index values obtained across all tests. For reference purposes, it also shows the values reported by the manufacturer and the emission limits permitted for this type of technology under the Mexican national standard NOM-42-SEMARNAT and EU standards. We recall that the emission indices reported by the manufacturer were obtained during lab tests on a chassis dynamometer following a homologation driving cycle. Therefore, the two values are not comparable because the values reported in our tests were obtained from the vehicle running under real driving conditions in this study. However, they can be used to confirm the values were within the same order of magnitude and the relevance of the test with PEMS under real driving conditions.

## 4. Discussion

The proposed method for the determination of the real mass emissions of CO_2_, CO, and NO_x_ in the tailpipe of fossil-fuel-powered vehicles by the use of low-cost sensors for the measurement of tailpipe concentrations, combined with a device to read the fuel consumption from the Engine Computer Unit (ECU), offers several key advantages.

### 4.1. Exactitude and Representativeness of the μPEMS Results

In the real world, instantaneous measurements of tailpipe emissions (in g/s) exhibit high variability due to uncertainty propagation in input variables and the inherent transients of driving. The RDE/PEMS literature converges on the view that representativeness is not achieved at the second-by-second scale but through statistical aggregation of micro-trips to obtain driving patterns, capturing how people drive in a region [68]. In practice, normalized emission indices (g/km) stabilize after sufficient distance and fuel consumption have been accumulated, using segment-weighted averages and cumulative metrics (with moving time windows and uncertainty estimates), or by reproducing controlled driving cycles that statistically capture usage regimes. This approach, grounded in real driving conditions and convergence analysis, yields robust, comparable emission factors suitable for regulatory assessment and field diagnostics.

*The CO_2_ sensor:* According to various studies [69,70,71,72], the most robust estimate of the CO_2_ EI (g/km) is obtained using a fuel-based approach—from the specific fuel consumption (SFC) and a conversion factor that depends on the fuel’s carbon content (carbon balance method). This approach reduces sensitivity to concentration noise and instrumental phase shifts and typically yields lower uncertainties under real driving conditions.

Nevertheless, the NDIR sensor included in the system provides diagnostic value: its temporal concentration profile in RDE enables the detection of operational deviations in the powertrain (e.g., anomalous transients, prolonged enrichments, aftertreatment inefficiencies) that have been documented in previous studies. Results showed that the NDIR signal qualitatively reproduces the trend of the reference PEMS; however, the coefficient of determination is *R*^2^ ≈ 0.67, indicating limited point-by-point agreement.

In summary, we propose employing the SFC-based method for the primary quantification of the CO_2_ EI and using the NDIR sensor as complementary observability for dynamic analysis and early fault detection at a very efficient cost.

### 4.2. Implications from the Industrial Perspective

From an operational standpoint, the μpems exhibits substantial flexibility. It can be applied during laboratory or on-the-road tests conducted under normal driving conditions. It can also be used with data collected by monitoring a vehicle without interfering with its operation.

In economic terms, the method is low cost compared with existing alternatives. It does not require laboratory infrastructure or formal testing protocols. It requires continuous monitoring at 1 Hz of the following operating variables: location, engine RPM, air–fuel ratio, vehicle speed, instantaneous fuel consumption, and the concentration of each pollutant gas (CO and NO_x_). This activity can be carried out using the instruments installed by vehicle manufacturers to control vehicle operation and the implementation of low-cost sensors for the measurement of tailpipe concentrations. Thus, it does not require high instrumentation costs comparable to those of PEMS or mini-PEMS. However, it requires a telemetry system that reads such data, aggregates it in a central computer, and processes it according to the proposed method. Currently, telemetry companies charge about USD 200 for installing their telemetry devices and USD 50 per vehicle per month for the monitoring service. These figures are substantially lower than the cost of a test using PEMS (~1 million USD of CAPEX and ~0.05 million USD of OPEX per test). We highlight that the development of the μPEMS prototype at TRL 3 or 4 has incurred significant costs, primarily for calibration and research. At a later stage (TRL 7–8), we will focus on manufacturing processes at an industrial scale that meet the low-cost requirement (<100 USD).

With respect to scalability, the proposed method is applicable to a wide range of transportation technologies that use internal combustion engines powered by fossil fuels, both in conventional propulsion configurations and in hybrid systems incorporating this type of engine. Consequently, its implementation is suitable for the analysis and evaluation of large-scale fleets.

### 4.3. Implications for Public Policy

In the current field of environmental technology, significant advances in cost-effectiveness and applicability have been achieved through the development of µPEMS designed for measuring pollutants in the exhaust systems of vehicles with internal combustion engines. The completion of this phase of the project has resulted in a system that combines a low-cost µPEMS to measure the mass flow of the main pollutants (CO, CO_2_, NO_x_) in gases emitted by internal combustion engines. These prototypes are affordable and easy to use, distinguishing them from current systems, which are typically expensive and complex to implement.

From a regulatory perspective, measuring the mass emission of pollutants under real-world driving conditions helps environmental authorities. All these advantages of the proposed method enable regulators to ensure that vehicles in circulation comply with environmental laws, holding manufacturers accountable for proper vehicle functioning and users for adequate maintenance. The method can help identify and promote options that remain within stipulated conformity limits and support the establishment of environmental performance standards, with a focus on environmentally friendly propulsion systems. However, its regulatory adoption will require standardization, metrological validation, and social acceptance.

The easy implementation of these µPEMS in aftertreatment systems not only enables effective monitoring and optimization but also ensures compliance with current environmental regulations. Owing to its low cost and considerable commercial potential, this patentable technology could catalyze a significant change in how manufacturers and regulatory bodies address environmental protection and air quality improvement. This technological development is a clear example of how targeted innovation not only contributes to environmental improvement, but also facilitates regulatory compliance [73,74], promotes the adoption of sustainable technological proposals to address the complex environmental problems caused by the automotive industry, and contributes to regulatory compliance.

### 4.4. Main Drawbacks of the Proposed Method for the Measurement of the Real Mass Emissions

For heavy and light duty vehicles, the measurement of real mass emissions faces mainly practical constraints: (a) cross-sensitivity and drift in MOX sensors, stemming from humidity and interferents such as HC, require thermal modulation and periodic recalibration to preserve selectivity and sensitivity; (b) synchronization and resampling are necessary to reconcile disparate channel frequencies (µPEMS/OBD/MOX), and decisions regarding harmonization, filtering, and temporal alignment influence bias and uncertainty; (c) maintenance and aging, through fouling and degradation of MOX/NDIR sensors and conditioning elements, demand routine verification and recalibration to prevent response shifts; and (d) achieving a unit cost below USD 100 for mass deployment depends on economies of scale, application-specific integrated circuits (ASICs), and optimized calibration processes, since otherwise the costs of materials, assembly, metrological assurance, housing, connectivity, and post-sale support may exceed this threshold and compromise economic viability.

### 4.5. Main Limitations of This Study

An important limitation of this study is that the experimental evaluation was conducted on only three vehicles. Even though the vehicles used represent different propulsion technologies (gasoline, diesel, and HEV), all are equipped with engines of approximately 2.5 L displacement and were manufactured after 2010. The restriction on the number of the μPEMS tests was due to the high costs associated with the tests, mainly associated with the use of NIST traceable calibration gases. Therefore, it is recommended that future research validate the µPEMS in vehicles across a wider range of engine displacements, ages, fuel types, and propulsion technologies.

### 4.6. Follow-Up Optimization Schemes for the Issues

The µPEMS faces significant limitations, including long-term drift in MOX sensors and sensitivity to temperature and humidity variations in exhaust gases. In this context, a promising line of research focuses on the development and application of equivalent vehicle maps, which enable estimation of pollutant emissions based on the vehicle’s energy consumption. As proposed in [75], these maps use operating parameters such as engine torque and revolutions per minute (RPM), which can be obtained reliably and at low cost. Furthermore, comparative analyses between different optimization approaches, including those based on these models, open the possibility of establishing more accurate and accessible methodologies for emissions assessment. Taken together, this approach could not only improve the accuracy of µPEMS but also promote their adoption in a wider range of applications and vehicle technologies.

## 5. Conclusions

The µPEMS is a low-cost device for the measurement of the real mass emissions of CO_2_, CO, and NO_x_ in the tailpipe of fossil-fuel-powered vehicles under real driving conditions. It consists of low-cost sensors for the measurement of tailpipe concentrations combined with a device to read the fuel consumption from the Engine Control Unit (ECU).

This manuscript presents the work carried out to develop a µPEMS. It deploys MOX sensors, which exhibit cross-sensitivity issues when measuring the concentration of CO and NO_x_ at the tailpipe conditions. We addressed this issue by using multiple linear regression models with temperature, humidity, pressure, and MOX readings. Under these circumstances, it demonstrated a robust correlation (*R*^2^ > 0.87) with experimental data obtained by testing gasoline, diesel, and hybrid vehicles under real driving conditions using a regulatory-compliant PEMS (AVL MOVES).

To convert these pollutant concentration measurements into mass emissions, we coupled them with readings of instantaneous fuel consumption taken directly from the ECU. Problems of varying sampling frequency and sensor time responses were solved by averaging independent variables with the same time window. Data time-alignment issues were addressed using dynamic data synchronization. Drift problems were mitigated by considering the time-accumulative results instead of instant variables.

Results were presented in terms of emission indices (g/km). When compared to results obtained by the well-accepted PEMS, it was found that the µPEMS monitors NO_x_ emissions with a high level of correlation in gasoline (*R*^2^ = 0.96), diesel (*R*^2^ = 0.95), and hybrid vehicles (*R*^2^ = 0.87). Similar results were obtained for CO.

This innovation can provide valuable data for improving vehicle technology and national emission inventories. It also enables new alternatives to regulate vehicular emissions. However, additional work is required to explore the possibility of using other sensors and to evaluate the performance of the µPEMS after long hours of service. Data drift is the main issue to be resolved.

## Figures and Tables

**Table 1:** Sensors to measure CO and NO_x_ at tailpipe conditions; * indicates methods approved by the USEPA.

Gas	Working Principle	Operating Conditions	Advantages	Disadvantages	Price USD	Size (Bore & Length)	Source
CO	NDIR *	Operating temperature: −40 to 85 °C.	High precision and selectivity. Fast response.	Sensitivity to humidity and temperature.	1165	9.13 × 17.5 mm	USEQGCDAC8L100 made by KEMET, Phoenix, USA [26]
	Electrochemical	Operating temperature: −20 to 80 °C. Relative humidity: 15–90% RH	Low consumption, wide linear range. Excellent repeatability and stability.	Limited useful life. Potential cross-sensitivity. Detection range: 0–10,000 ppm.	N/A	16.7 × 10.8 mm	CO sensor ME2-CO-Φ14 × 5 made by Winsen, Zhengzhou, China [27]
CO, CH_4_	MOS sensitivity CO, CH_4_	Operating temperature: −10 to 50 °C. Relative humidity: less than 95%RH	Low-cost. Small sizes. Long lifespan.	Detection range: 50–1000 ppm CO, and 300–10,000 ppm CH_4_. Exposure to corrosive gases, such as SO_x_, reduces its sensitivity. Keep them unused for a long time.	1.8	9.4 × 7.5 mm	MP-9 CO, CH_4_ semiconductor made by Winsen, Zhengzhou, China [28]
NO_x_	Amperometric double chamber principle	Operating temperature: 0 to 850 °C.	Detection range: 0–3000 ppm. High lifetime: 15,000 h.	Medium cost.	560	20 × 80 mm	EGS-NX made by Bosch, Gerlingen-Schillerhöhe, Germany [29]
	Chemiluminescence detection (heated) *	Ambient temperature: 5–40 °C. Humidity: under 80%RH	Detection range: 10–10,000 ppm. High accuracy. High selectivity. High durability.	Expensive. Requires frequent calibration.	>100,000	508(W) × 690(D) × 143(H) mm	MEXA-1170HCLDA made by HORIBA, Kyoto, Japon [30]
	MOS	Operating temperature: −40 to 125 °C.	Detects multiple gases, such as CO, NO, and NH_3_. Preheating time: 30 s.	Detection range: CO 1~5000 ppm, NO_x_ 0~10 ppm, and NH_3_ 1~300 ppm.	37	N/A	ZMHS10 semiconductor made by Winsen, Zhengzhou, China [31]
	MOX-Nanoz	Operating temperature: 0 to 80 °C.	Detection range: NO_x_ (0–3000 ppm), and CO (0–50,000 ppm). Small size and low power consumption.	Expensive.	300,000	N/A	Made by Nanoz, Rousset, PACA, France [32]
	MOX-UST (Umweltsensortechnik)	Operating temperature: 0 to 150 °C for a short time.	Detects multiple gases, such as CO, CH_4_, C_3_H_8_, and NO_2_. Economic, small, and does not need continuous calibration. High durability (up to 10 thousand hours).	Cross-sensitivity issues. Takes about 10 min to reach proper operating temperature.	110	8 × 24 mm	3A4P-UST Triplesensor made by UST Geratal Germany [33]

**Table 2:** Technical characteristics of the vehicles used for the on-road tests. Sources: [54,55,56,57].

Model	Mitsubishi L200/4WD/4CIL	Nissan NP300/4 × 2/4CIL	Toyota RAV 4 Hybrid 22H/4 × 4/4CIL
Model year	2010	2019	2023
Type of vehicle	Pick-up	Pick-up	SUV
Engine model	Not available	QR25	A25A-FXS
Compression ratio	17.5:1	10:1	14:1
Displacement	2500 cm^3^	2500 cm^3^	2500 cm^3^
Max power	100 KW at 4000 rpm	122.1 KW at 6000 rpm	130 kW at 6000 rpm
Max torque	314 Nm at 2000 rpm	241.33 Nm at 4000 rpm	221 Nm at 3600–5200 rpm
Fuel	Diesel cetane 45	Gasoline octane 92	Gasoline octane 87
Exhaust certification	EURO 4	NOM-042-SEMARNAT-2003	EURO 6d
Driving cycle	On-road	On-road	On-road

**Table 3:** Technical characteristics of the measurement systems installed on the tailpipes of vehicles to measure the real mass emissions of vehicles. Sources: [29,33,58,59,60,61,62,63,64].

Measurement System	Operating Principle	Variable to be Measured	Range	Accuracy
AVL MOVE iS+ PEMS made by AVL List GmbH, Graz, Austria	NDIR	CO_2_	0 to 20%	±2% relative
	NDIR	CO	0 to 5%	±2% relative
	NDUV	NO	0 to 5000 ppm	±2% relative
	NDUV	NO_2_	0 to 2500 ppm	±2% relative
	Pitot tube	Exhaust flow meter	50…2200 kg/h	±2% of reading or ±0.5% of full scale, whichever is greater
	Photoacoustic measurement & gravimetric filter module	Particulate matter (PM)	1000 mg/m^3^	1 µg/m^3^
MOX-µPEMS	Multiple metal oxide semiconductors	CO	50…3500 kΩ	N/A
		NO_2_	30…3000 kΩ	N/A
		CH_4_, C_3_H_8_	30…3500 kΩ	N/A
	CMOSens, SHT41 made by Sensirion AG, Stäfa, Switzerland	Humidity Temperature	0 to 100%RH −40 to 125 °C	±1.8%RH ±0.2 °C
	Ultra-compact piezoresistive, LPS22HB made by ST Arizona, USA	Pressure Temperature	26 to 126 kPa −40 to 125 °C	±0.1 kPa ±0.2 °C
NO_x_-µPEMS	Amperometric double-chamber principle, Bosch NO_x_ sensor EGS-NX2	NO_x_	0 to 1650 ppm	±10 ppm new/±12 ppm used
	Planar ZrO_2_ dual cell limiting current sensor, Bosch LSU4.9 UEGO sensor	Air/fuel ratio	Lambda 0.65 to ∞, gasoline or diesel automotive engine	±0.7%
Type-K Thermocouple made by Analog Devices Wilmington, MA, USA	Seebeck effect	Exhaust gases temperature	0 to +1024 °C	0.25 °C
OBD made by Elm Electronics, Ontario, CAN	Inventure CAN reader, ELM 327	Raw CAN bus data	N/A	N/A

**Table 4:** Determination coefficients obtained by predicting the NO_x_ concentration using Equation (6) in road tests with diesel, gasoline, and hybrid gasoline–electric vehicles. Note: _1_: response of the heated MOX sensor at 350 °C; _2_: response of the heated MOX sensor at 425 °C.

Regression Statistics						
	Diesel Vehicle		Gasoline Vehicle		HEV	
Calibration Version	NO_x_-µPEMS		NO_x_-µPEMS		MOX-µPEMS	
Multiple R	0.977		0.980		0.932	
R-Square	0.955		0.961		0.87	
Adjusted R-Square	0.899		0.764		0.682	
Standard Error	12.315		13.758		4.032	
Observations	19		13		18	
	Coefficient	*p*-Value	Coefficient	*p*-Value	Coefficient	*p*-Value
Intercept	6715.776	0.245	−15,642.29	0.306	−342.359	0.343
RH%	20.602	0.170	2.33	0.370	0.094	0.743
RH% Temperature	12.481	0.096	35.38	0.276	0.352	0.917
Pressure Temperature	−4.508	0.340	−24.67	0.318	0.322	0.924
Pressure Absolute	−7.953	0.156	15.91	0.310	0.317	0.364
ln (CH/CHref) _1_	−0.057	0.098	−0.13	0.992	4.024	0.166
ln (CO/COref) _1_	−0.009	0.181	−0.84	0.694	1.183	0.677
ln (NO_x_/NO_x_ref) _1_	0.003	0.098	5.10	0.099	11.430	0.293
ln (CH’_s_/CH’_s_ref) _2_	−0.301	0.722	−21.99	0.186	13.030	0.070
ln (CO/COref) _2_	−0.008	0.946	3.76	0.149	−10.868	0.022
ln (NO_x_/NO_x_ref) _2_	0.104	0.056	−1.89	0.079	−11.289	0.084

**Table 5:** CO and NO_x_ emission indices obtained by RDE tests for diesel, gasoline, and HEVs tested in this study. ^a^: Euro 4 emission standards, for compression ignition (diesel); N_1_, class III > 1760 kg. ^b^: Euro 7 emission standards, for positive ignition (gasoline); N_1_, class III > 1760 kg. ^c^: Euro 7 emission standards, for positive ignition (gasoline); N_1_, class II (1305–1760) kg. ^d^: Maximum emission limits for light-duty vehicles under the National Regulation Standard C (NOM-042-SEMARNAT-2003). Sources: [56,65,66,67].

Source		NO_x_ (mg/km)		CO (mg/km)
	Diesel	Gasoline	HEV	HEV
Monitored with AVL MOVE iS+ PEMS	N/A	N/A	10.4	470
Monitored with MOX-µPEMS	7017	83.8	10.5	483
Monitored with NO_x_-µPEMS	7595	83.6	11.0	N/A
Manufacturer-provided data for compliance certification	487	113.0	11.4	457
National regulations	250 ^d^	80.0 ^d^	80.0 ^d^	1000 ^d^
Maximum emission limits for LDVs under EU standards	390 ^a^	82.0 ^b^	75.0 ^c^	1810 ^c^

## Data Availability

The data presented in this study are available from the corresponding authors upon request.

## Abbreviations

The following abbreviations are used in this manuscript:

μpems	Micro-PEMS
CMOSens	Complementary Metal Oxide Semiconductor
ECU	Engine Computer Unit
EI	Emission Index
MOX	Metal Oxide Sensor
NDIR	Non-Dispersive Infrared
NDUV	Non-Dispersive Ultraviolet
N/A	Not Available
OBD	On-Board Diagnostics System
PEMS	Portable Emissions Measurement System
RDE	Real Driving Emissions
WLTC	Worldwide Harmonized Light Vehicles Test Cycle
WLTP	Worldwide Harmonized Light Vehicles Test Protocol
